# Production, purification, characterization and application of two novel endoglucanases from buffalo rumen metagenome

**DOI:** 10.1186/s40104-022-00814-z

**Published:** 2023-02-06

**Authors:** Zhenxiang Meng, Chengjian Yang, Jing Leng, Weiyun Zhu, Yanfen Cheng

**Affiliations:** 1 Laboratory of Gastrointestinal Microbiology, National Center for International Research On Animal Gut Nutrition, Nanjing Agricultural University, Nanjing, 210095 China; 2 Buffalo Research Institute, Chinese Academy of Agricultural, Nanning, 530000 China; 3 Key Laboratory of Animal Nutrition and Feed Science of Yunnan Province, Yunnan Agricultural University, Kunming, 650000 China

**Keywords:** Endoglucanase, In vitro, Lignocellulosic substrates, Rumen fermentation

## Abstract

**Background:**

Lignocellulose biomass is the most abundant and renewable material in nature. The objectives of this study were to characterize two endoglucanases TrepCel3 and TrepCel4, and determine the effect of the combination of them (1.2 mg TrepCel3, 0.8 mg TrepCel4) on in vitro rumen fermentation characteristics. In this study, three nature lignocellulosic substrates (rice straw, RS; wheat straw, WS; leymus chinensis, LC) were evaluated for their in vitro digestibility, gas, NH_3_-N and volatile fatty acid (VFA) production, and microbial protein (MCP) synthesis by adding enzymatic combination.

**Methods:**

Two endoglucanases’ genes were successfully expressed in *Escherichia coli* (*E. coli*) BL21 (DE3), and enzymatic characteristics were further characterized. The combination of TrepCel3 and TrepCel4 was incubated with lignocellulosic substrates to evaluate its hydrolysis ability.

**Results:**

The maximum enzymatic activity of TrepCel3 was determined at pH 5.0 and 40 °C, while TrepCel4 was at pH 6.0 and 50 °C. They were stable over the temperature range of 30 to 60 °C, and active within the pH range of 4.0 to 9.0. The TrepCel3 and TrepCel4 had the highest activity in lichenan 436.9 ± 8.30 and 377.6 ± 6.80 U/mg, respectively. The combination of TrepCel3 and TrepCel4 exhibited the highest efficiency at the ratio of 60:40. Compared to maximum hydrolysis of TrepCel3 or TrepCel4 separately, this combination was shown to have a superior ability to maximize the saccharification yield from lignocellulosic substrates up to 188.4% for RS, 236.7% for wheat straw WS, 222.4% for LC and 131.1% for sugar beet pulp (SBP). Supplemental this combination enhanced the dry matter digestion (DMD), gas, NH_3_-N and VFA production, and MCP synthesis during in vitro rumen fermentation.

**Conclusions:**

The TrepCel3 and TrepCel4 exhibited the synergistic relationship (60:40) and significantly increased the saccharification yield of lignocellulosic substrates. The combination of them stimulated in vitro rumen fermentation of lignocellulosic substrates. This combination has the potential to be a feed additive to improve agricultural residues utilization in ruminants. If possible, in the future, experiments in vivo should be carried out to fully evaluate its effect.

**Supplementary Information:**

The online version contains supplementary material available at 10.1186/s40104-022-00814-z.

## Introduction

Lignocellulosic biomass is the most abundant, inexpensive, available and renewable raw material in nature for industrial biorefining and livestock farming [1, 2]. Lignocellulosic biomass is conversed into a variety of bio-based products (papers, textiles, animal feed stocks) and bio-energy (bioethanol) by industrial biorefining [3, 4]. Over 200 value-added chemicals and polymers are derived from lignocellulosic biomass using biorefining [5]. The lignocellulosic biomass mainly includes agricultural residues, urban wood waste/mill residues, forestry residues and energy crops, which principally consists of 35% to 50% cellulose, 20% to 35% hemicellulose, and 5% to 10% lignin [5]. Among the agricultural residues, rice straw (RS) and wheat straw (WS) and corn straw (CS) with more than 70 million tons every year are the main sources for reducing sugars [6]. In addition, agricultural residues are thought to be a valuable feedstuff for ruminants because of considerable quantities. However, most of them are randomly discarded or burned, resulting in resource wasting and environmental pollution. Although this agricultural straw fiber can be decomposed with ruminal microbiota, their ruminal degradability is still relatively low [7]. To enhance this straw fiber digestibility in rumen, adding exogenous fibrolytic enzymes (EFEs) with ruminant diets has been widely studied [8–10].

Cellulases are grouped into 17 glycoside hydrolase (GH) families based on the sequence and structure similarity of CAZy database (Carbohydrate-Active Enzyme, http://www.cazy.org), including GH5 to 10, 12, 26, 44, 45, 48, 51, 60, 61, 74, 124 and 131 [11]. Among these, GH5 is the largest family, and most of the GH5 cellulases are endoglucanases [11, 12]. Endoglucanase is more effective than exoglucanase in modifying the properties of cellulose fibers, which could not damage fiber properties due to the lack of activity on crystalline cellulose [13]. Because of such specific characteristics, endoglucanase was widely applied in industrial biorefining and livestock farming, especially in the production of fermentable sugar to be converted to bioethanol [14] and volatile fatty acid (VFA) [15]. Several novel and efficient endoglucanases had been directly isolated from various environments such as decomposed leaves/woods [16], insects [17], lake sediment [18], acidic hot springs [19] and animals [20]. Among animals, the rumen of herbivores, such as moose [21], sheep [22], gayals [23], buffaloes [24], cows [25] and camels [26], is capable of hydrolyzing cellulose and other complex polysaccharides by lignocellulosic enzymes secreted by microbes. However, major group of microbes in rumen cannot be cultivable or isolated in vitro. With the application of metagenomic or metatranscriptomic screening approaches, desired CAZy from rumen microbiome were identified and analyzed [27]. However, a large portion of candidate CAZy were not cloned and comprehensively characterized until now.

The bio-degradation of lignocellulosic biomass is a complex process consists of these steps: pretreatment of biomass for enzymatic hydrolysis of the polysaccharides, and fermentation of fermentable sugars to bioethanol or VFA. Hence, cellulases with high hydrolytic ability are essential to decrease the loading amounts of enzymes, which could decrease economic cost [28]. Enzymatic cocktails work synergistically is a critical step to degradation polysaccharides and crystalline cellulose [29]. Enzymatic combinations could attain enhanced stability at elevated temperatures [30] and broad range pH value ranges [31]. Recently, for bioconversion process, some researchers have utilized enzymatic combinations to enhance the biocatalytic productivity and saccharification of lignocellulose [32–35]. The combination of endoglucanase, β-glucosidase and xylanase can efficiently hydrolyze lignocellulosic substrates. For instance, Agrawal et al. [35] presented that endoglucanase, β-glucosidase and xylanase at the ratio of 20.40:38.43:41.16 could maximize the saccharification yield from the steam explosion of wheat straw. To improve the production of bioethanol, Soleimani and Ranaei-Siadat [36] optimized the influential factors and obtained the best ratio of exoglucanase, endoglucanase and β-glucosidase (1:5:1, mg/g bagasse). Jain et al. [37] found that a stable mutant endoglucanase UV-8 of *Talaromyces verruculosus* IIPC 324 with a concentrated fungal enzyme (CFE) regarded as the best cellulase cocktail, which can produce beyond three times fermentable sugar than that of Palkonol MBW. Zhao et al. [38] presented that two neutral thermostable cellulases from *Phialophora* sp. G5 showed synergistical effect on the hydrolysis of filter paper. The saccharification yield could be improved by enzymatic cocktails contain lytic polysaccharide monooxygenases (LPMOs) that leave cellulose using molecular oxygen and an electron donor [29, 39]. For livestock farming, enzymatic combinations were also widely applied to improve ruminal fermentation and degradability [40, 41]. Bowman et al. [40] showed that the addition of commercial xylanases and cellulases together provided by Promote N.E.T. (Agribrands International, St. Louis, MO, USA) to the concentrate portion of the TMR consisting of 45% of the dietary (dry matter, DM) could increase milk production of lactating dairy cows. Yang et al. [41] presented that two enzyme additives (CE14, CE24) showed greatly positive effects on DM, neutral detergent fiber (NDF) and acid detergent fiber (ADF) degradability of alfalfa hay. Although many commercial enzymatic combinations can play an important role in bio-degradation and livestock farming, most of them have low enzymatic activities, and are not purified. Therefore, these two factors make it difficult to fully evaluate the effect of enzymatic combinations.

The buffalo diet consists of a variety of recalcitrant lignocellulosic materials including aquatic plants, crops, grasses, herbs, microalgae, leaves and bark of trees [42]. Therefore, the investigation of key enzymes in buffalo rumen will enhance understanding of plant biomass digestion, which could be useful for industrial bioconversion and livestock farming processes [43]. We hypothesized that the endoglucanases from buffalo rumen metagenome can be successfully expressed and hydrolyze natural lignocellulosic substrates in vitro. Hereby, this study presents the expression and characterization of two novel endoglucanases with high catalytic ability from buffalo rumen microbial metagenome. The TrepCel3 and TrepCel4 were optimally active at acidic pH conditions, and TrepCel4 showed its maximum activity at 50 ℃. The TrepCel3 and TrepCle4 could efficiently hydrolyze agricultural residues, but the combination of them had a superior ability to maximize the saccharification yield. The RS, WS, leymus chinensis (LC) and sugar beet pulp (SBP) were also used to evaluate this combination’s ability to improve in vitro rumen fermentation.

## Materials and methods

### Strains, vector, and media

*Escherichia coli* (*E. coli*) DH5α (Vazyme, Nanjing, China) was used for the propagation of plasmid and *E. coli* BL21 (DE3) (Tsingke, Beijing, China) was used for the expression of recombinant product. Plasmid pET-28a (+) (Novagen, Madison, USA) was used for In-Fusion cloning, and expression of recombinant protein. Carboxymethylcellulose (CMC), locust bean gum (LBG), lichenan, barley β-glucan, filter paper (Whatman Grade 40), *p*-nitrophenyl-β-*D*-glucopyranoside (*p*NPG) were purchased from Megazyme (Bray, Wicklow, Ireland), General Electric Company (Boston, Massachusetts, USA) and Sigma-Aldrich (Kenilworth, New Jersey, USA). The RS, WS, LC and SBP were purchased from the local market (Nanjing, Jiangsu, China).

### Ruminal in sacco incubations

Three healthy, mature and rumen-cannulated buffaloes (Nanning, Guangxi, China) were used in this study. The buffaloes were fed chopped green roughage ad libitum for one month, and had free access to drink fresh water. All animal procedures were performed in accordance with Institutional Animal Care and Use Committee of Nanjing Agricultural University (GB14925, NJAU-CAST-2011–093). A total of 6 stitched nylon bags were filled with wheat straw (2.5 g/bag, DM) were placed into the buffalo rumen (2 bags/buffalo). All bags were retrieved at 24 h, followed by washing gently with phosphate buffered saline (PBS) buffer to remove the rumen contents attached to the bag and loosely attached microbes. The collected sample residues were frozen immediately in liquid nitrogen, and stored at − 80 °C until DNA isolation.

### Identification of endoglucanase enzyme sequences

Genomic DNA was extracted using the protocol as Cheng et al. [44] described. The quantity and the integrity of DNA extracted were determined using Invitrogen Qubit 4 (Thermofisher, Massachusetts, USA) and 1.2% gel electrophoresis, respectively. The metagenome library was prepared using the Nextera DNA Library Preparation Kit (Illumina, CA, USA) under the manufacturer’s protocol. The quantity assessment of the library was performed using the Agilent 2100 Bioanalyzer System (Agilent Technologies, CA, USA). The small DNA fragment library (300 to 400 bp) was further sequenced at the Beijing Genome Institute (BGI, Shenzhen, China). High-quality reads of buffalo rumen metagenome data were obtained after quality control by FastQC, followed by assembled de novo into contigs using IDBA-UD v1.1.1 [45] and SOAP2 software [46]. The distribution of high-quality reads was alignment to the construction of reference gene sets by Bowtie2 [47]. The predicted gene sets were functionally annotated against the CAZy database using the blast software (E-value < 0.00001). According to sequence alignment, those protein sequences with the highest similarity were obtained, and the corresponding protein function annotations were also confirmed. Among predicted gene sequences, those confirmed as cellulases by CAZy database were selected for further studied. Finally, two of those sequences that passed all filters, named TrepCel3 and TrepCel4, respectively, were selected for experimental assays. The metagenomic data was submitted to the NCBI Sequence Reads Archive (SRA) as BioProject: PRJNA815894.

### Bioinformatic analyses

The sequences of TrepCel3 and TrepCel4 were submitted to NCBI GenBank (No. OM986951, No. OM986952). The TrepCel3 and TrepCel4 sequences were both synthesized by Sangon Biotechnology (Shanghai, China). The analyses of conserved domain were performed using the NCBI conserved domain database (CDD) (https://www.ncbi.nlm.nih.gov/Structure/cdd/wrpsb.cgi). The analysis of signal peptides was carried out by using the SignalP-6.0 online tool [48]. Multiple sequence alignment was conducted by the online web tool of Clustal Omega (https://www.ebi.ac.uk/Tools/msa/clustalo/). The phylogenetic tree was further constructed by MEGA-X according to the neighbour-joining method [49]. The calculated molecular weight (Mw) and isoelectric point (pI) were predicted by the Expasy online tool [50]. The enzyme domain functional analysis was conducted by InterProScan (http://www.ebi.ac.uk/interpro/search/sequence/). The homology modelling and secondary structure of these two candidate enzymes were done predicted by using the Phyre2 online tool [51], and the candidate tertiary structure with 100% confidence score and the highest coverage score was further chosen.

### Recombinant protein expression and purification

Recombinant *E. coli* BL21 (DE3)/pET28a (+)/TrepCel3, and BL21 (DE3)/pET28a (+)/TrepCel4 were cultured in 6 mL Luria–Bertani (LB) medium containing 50 μg/mL kanamycin at 37 °C with shaking at 200 r/min overnight. The growing cells were then inoculated into in a 1-L flask containing 600 mL LB medium supplemented with 50 μg/mL kanamycin and incubated at 37 °C with shaking at 200 r/min. When the optical density at 600 nm of the culture reached 0.5 to 0.6, a final concentration of 0.5 mmol/L isopropyl β-*D*-thiogalactopyranoside (IPTG) was added, and then incubated for 24 h further in 16 °C, 150 r/min shaking conditions. Later, the cells were collected by centrifugation at 4 °C and 12,000 r/min for 30 min. The pellets were further resuspended in 40 mL of lysis buffer (50 mmol/L NaH_2_PO_4_, 300 mmol/L NaCl, 10 mmol/L imidazole, pH 8.0), disrupted by sonication (400 W, 30 min), followed by centrifugation at 12,000 r/min for 15 min at 4 °C to obtain the supernatant. The collected supernatant was purified by Ni–NTA Fast Start Kit using standard protocols (Qiagen, Hilden, Germany). Briefly, the cell lysate supernatant was applied into a Fast Start Column with 6xHis-tagged resin. The bound 6xHis-tagged protein was eluted by Native Elution Buffer (250 mmol/L imidazole, 300 mmol/L NaCl, pH 8.0). The quality and concentration of the purified fractions were measured by 12% SDS-PAGE gel, and Bradford method [52], respectively.

### Cellulase activity assay and characterization

Cellulase activity was measured by the DNS method with CMC as a substrate [53]. The release of reducing sugar was quantified through the spectrophotometric method. The cellulose substrate CMC was used in the enzymatic characterization for all experiments in this study. Briefly, 50 μL protein was incubated with 150 μL of 2% (w/v) CMC at 40 °C and pH 7.0 for 5 min, followed by addition of 300 μL DNS and boiling for 5 min. After cooling room temperature (RT), the reaction mixture was diluted and then measured at 540 nm using a TECAN Spark Multi-functional Microplate Reader (Laubisrütistrasse, Zurich, Switzerland). One unit (U) of cellulase activity was defined as the amount of enzyme that liberate 1 μmol of reducing sugar per minute under the assay conditions. For cellulase activity assay, each treatment was incubated in triplicate.

To determine the pH optima of enzymatic activity, purified TrepCel3 and TrepCel4 were incubated with 2% (w/v) CMC in various pH buffers, respectively. The buffers used were 100 mmol/L citric acid-Na_2_HPO_4_ (pH 3 to 7), 100 mmol/L Tris–HCl (pH 8 to 9) and 100 mmol/L glycine–NaOH (pH 10 to 12). The pH stability was evaluated by pre-incubating the enzyme at 4 °C for 16 h at different pH buffers, and then the residual activity was measured. The enzyme solution in 100 mmol/L citric acid-Na_2_HPO_4_ buffer with 2% (w/v) CMC was incubated in the different temperature (30 to 70 °C) for 10 min to determine the optimum temperature. The thermal stability was used by observing the residual enzyme activity after incubation of the enzymes in 100 mmol/L citric acid-Na_2_HPO_4_ buffer at 50 and 60 °C for 60 min without substrate. The residual enzyme activity was determined at different time intervals (0, 10, 20, 30, 40, 50 and 60 min). All assays were measured by the DNS method at 540 nm, and the maximum enzyme activity was established as 100%.

The effect of 10 mmol/L K^+^, Cu^2+^, Mg^2+^, Zn^2+^, Ca^2+^, Mn^2+^, Fe^2+^, Fe^3+^, Co^2+^, Ni^2+^, NH_4_^+^ and Ba^2+^, 10 mmol/L PMSF, EDTA and β-mercaptoethanol, 1% (w/v) Tween 20, Tween 80, Triton X-100 and SDS, 2 mmol/L urea and guanidine hydrochloride on the enzymatic activity was measured in individual reactions by pre-incubating with enzyme for 60 min at RT using the DNS method [54–56]. The enzymatic activity in the absence of any additional reagent was taken as 100%, and defined as control.

The NaCl resistance test of TrepCel3 and TrepCel4 was determined by different concentrations of NaCl (1 to 5 mmol/L); without adding NaCl was set as the control group [57]. Besides, to determine the NaCl stability, the purified enzymes were placed in 1 to 5 mmol/L NaCl for 60 min at RT, and the residual enzyme activity was measured by the DNS method at optimum temperature and pH value [58]. The enzymatic activity in the absence of NaCl reagent was taken as 100%, and defined as control. To investigate the potential application of TrepCel3 and TrepCel4 for hydrolysis in seawater, enzymatic activities of them were further conducted in artificial seawater composed of NaCl, 26.29 g/L; CaCl_2_, 0.99 g/L; MgCl_2_·6H_2_O, 6.09 g/L; KCl, 0.74 g/L; MgSO_4_·7H_2_O, 3.94 g/L [57].

### Substrate specificity and kinetic parameters

The substrate specificity of purified cellulase was determined in the reaction mixtures with CMC, β-glucan, filter paper, LBG, lichenan, α-*p*NPG and β-*p*NPG. The enzymatic activity was determined by measuring the release of reducing sugar or *p*-nitrophenol (*p*NP) from various substrates [53, 59]. The reaction system contained 50 µL of purified enzyme and 150 µL of citric acid-Na_2_HPO_4_ buffer containing 2% (w/v) cellulose substrate, or 2 mmol/L *p*NPG at individual optimal pH and temperature for 5 min. Then 300 μL of DNS solution was added to end the reaction and boiled for 5 min, or the reaction mixture was stopped with 400 μL of 2 mmol/L Na_2_CO_3_. The released reducing sugar or *p*NP was measured as absorbance at 540 or 405 nm by using a TECAN Spark Multi-functional Microplate Reader (Laubisrütistrasse, Zurich, Switzerland), respectively. One unit of cellulase activity is defined as the amount of TrepCel3 or TrepCel4 producing 1 μmol of *p*NP per min per milliliter. For the kinetic parameters test of TrepCel3 and TrepCel4, *K*_m_ and *V*_max_, were measured with the substrate (β-glucan, lichenan and CMC) between 1 and 20.0 mg/L in citric acid-Na_2_HPO_4_ buffer by the Lineweaver–Burk method for 5 min. Each treatment was incubated in triplicate.

### Hydrolysis of nature lignocellulosic substrates

The synergistic effect of TrepCel3 and TrepCel4 in the degradation of lignocellulosic substrates was further studied. Therefore, the effect of different ratios of TrepCel3 and TrepCel4 (100:0, 80:20, 60:40, 40:60, 20:80, 0:100) on the hydrolysis of CMC was firstly studied [31]. Hence, 10 μL (0.5 mg/mL) of enzyme solution was incubated in 1.5 mL centrifuge tube with 90 µL of 2% CMC solution at 40 ℃ for 5 min, and then the mixture reaction was diluted, and measured by DNS method. Each treatment was incubated in triplicate.

The RS, WS, LC and SBP were evaluated for the hydrolysis capabilities in lignocellulose biorefinery. The RS, WS and LC were grinded to the particle size of 2 to 3 mm, and SBP was milled and sieved through a 50-mesh sieve. These lignocellulosic substrates were treated by 2% NaOH treatment at 121 °C for 15 min, and then washed with distilled water to fully remove reducing sugars. The purified 50 μL (0.5 mg/mL) TrepCel3, TrepCel4 and the combination of them were incubated with the substrates (2%, w/v) soaked in 20 mL citric acid-Na_2_HPO_4_ buffer, respectively. Each treatment was incubated in triplicate, and was in an incubator shaker at 60 r/min at 45 °C for 168 h [55]. The production of reducing sugar was measured using the DNS method at 3, 6, 14, 24, 48, 72, 96, 120, 144 and 168 h, respectively.

### In vitro rumen fermentation

The effect of the combination of TrepCel3 and TrepCel4 on rumen microbial fermentation of RS, WS and LC was investigated by an in vitro experiment. Rumen fluid was collected from three ruminally-fistulated Hu sheep (Mean ± SD, 65 ± 3.0 kg) fed a diet consisting of 700 g/kg alfalfa and 300 g/kg concentrates before morning feeding. Rumen samples were then squeezed through four layers of sterile cheesecloth. The in vitro study was performed in 120-mL bottles containing 600 mg agricultural straws, and 60 mL buffered rumen fluid that was composed of 45 mL buffer solution and 15 mL filtered rumen fluid [60]. The combination of these two endoglucanases (1.2 mg TrepCel3, 0.8 mg TrepCel4) was added into the bottles as treatments, and the same amount of PBS buffer instead of enzyme was incubated similarly as controls [61]. Each treatment had triplicate replicates and a blank control to correct for the gas production. All sample bottles were incubated in at 39 °C for 48 h with 60 r/min by using a Double-Layer Shaking Incubators ZWYR-4912 (Zhicheng Analytical Instrument Manufacturing Co., Ltd., Shanghai, China). The gas production was measured using a based on the pressure transducer technique (PTT) [62] at 48 h, and the pH was determined using the Seven2Go advanced single-channel portable pH meter (Mettler Toledo, Zurich, Switzerland). Procedures for analysis of VFA, NH_3_-N and microbial protein (MCP) were described by Weatherburn [63], Carro et al. [64], and Makkar et al. [65], respectively. In brief, VFA production was measurement by Agilent 7890B gas chromatography (Stevens Creek Blvd, California, USA), and NH_3_-N, MCP and lactate concentration was measured by using a TECAN Spark Multi-functional Microplate Reader (Laubisrütistrasse, Zurich, Switzerland) based on the spectrophotometer method. The residue was filtered using nylon bags for the determination of DMD.

### Statistical analyses

Statistical analysis was done by the one-way ANOVA with LSD multiple range test was performed to evaluate the significance of the hydrolysis of RS, WS, LC and SBP. Independent samples *t*-test was conducted to determine the results of in vitro rumen fermentation experiment. IBM SPSS Statistics 20 (Chicago, IL, USA) was used for statistical analysis. Significance was defined as *P* < 0.05.

## Results and discussion

Based on the metagenome data for identifying new endoglucanases with valuable industrial and livestock farming applications, in this study, two novel endoglucanases were mined from the metagenome data of buffalo rumen using computational screening, and named TrepCel3 and TrepCel4, respectively. After the in-silico screening, TrepCel3 and TrepCel4 were cloned, expressed, purified and characterized.

### In-silico sequence analysis of the putative cellulases

**Fig. 1:**
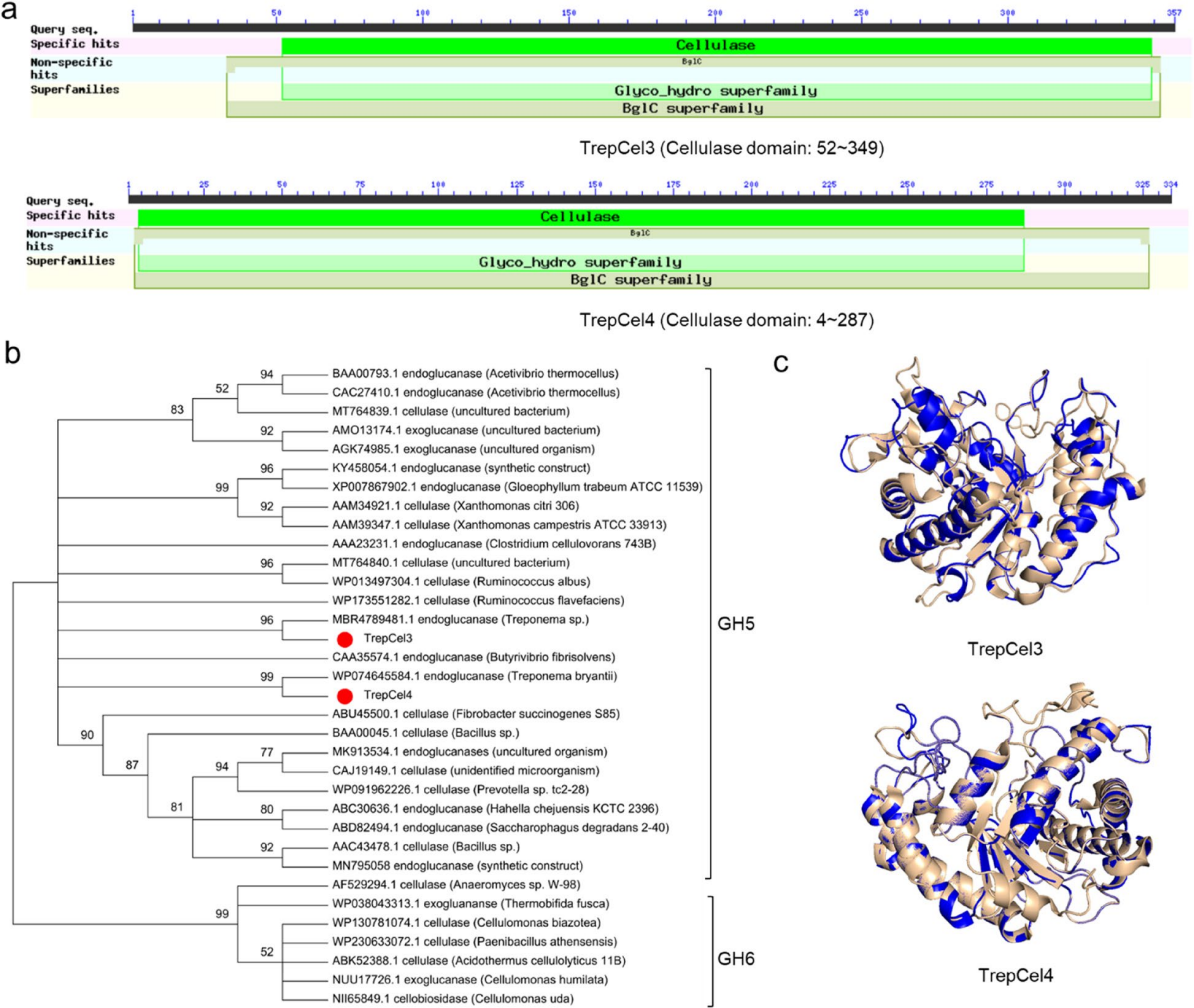
Structural analysis of TrepCel3 and TrepCel4. **a** Graphical summary of cellulase conserved domain; **b** Phylogenetic analysis of TrepCel3 and TrepCel4; **c** 3D structures of TrepCel3 and TrepCel4

The full open reading frame (ORF) length of TrepCel3 consisted of 1131 base pairs (bp), and the N-terminal 19 amino acids were predicted to be a putative signal peptide. TrepCel3 encoded a polypeptide of 357 amino acids with a theoretical molecular weight (Mw) of 39.8 kDa and isoelectric point (pI) 5.04, and only contained a module domain (52 to 349 amino acids; Fig. 1a). The amino acid sequence of TrepCel3 showed up to 82.63% identity with an endoglucanase from *Treponema* sp. based on the non-redundant (NR) protein database (GenBank: MBR4789481.1), while showed up to 52.6% identity with an endoglucanase from *Treponema bryantii* based on UniProt database (Accession: A0A1H9EK09). The results of phylogenetic tree showed that TrepCel3 was classified into the GH5 family (Fig. 1b). The results of Phyre2 showed that the most similar tertiary structure to TrepCel3 was an endoglucanase from *Caldicellulosiruptor* sp. f32 (PDB: 4X0V) with 100% confidence and 92% coverage (Fig. 1c). The predicted secondary structure of TrepCel3, contained 33% α-helix and 12% β-strand (Additional file 1: Fig. S1a).

The 1005-bp full ORF length of TrepCel4 encoded a protein of 334 amino acids with a calculated Mw of 37.9 kDa and pI 4.86. Deduced TrepCel4 had no signal peptide and also only contained a module domain (4 to 287 amino acids; Fig. 1a). The amino acid sequence of TrepCel4 was 81.63% identical to a characterized endoglucanase from *Treponema bryantii* (GenBank: WP074645584.1) based on NR protein database, while showed up to 81.0% identity with an endoglucanase from *Treponema bryantii* based on UniProt database (Accession: A0A1H9JN96). As shown in Fig. 1b, TrepCel4 was classified into GH5 family. The most similar tertiary structure to TrepCel4 was an endoglucanase from *Butyrivibrio proteoclasticus* (PDB: 4NF7). The predicted secondary structure of TrepCel4, contained 35% α-helix and 12% β-strand (Additional file 1: Fig. S1b).

### Expression, purification of recombinant enzymes

**Fig. 2:**
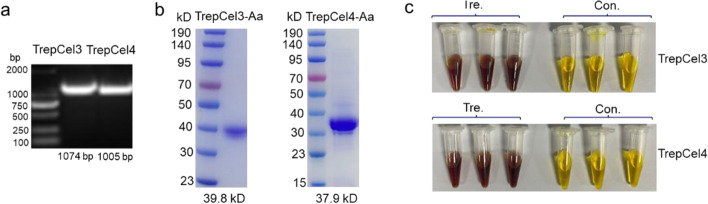
Analysis of purity and functional activity of TrepCel3 and TrepCel4. **a** The location of TrepCel3 and TrepCel4 genes; **b** SDS–PAGE analysis of purified recombinant TrepCel3 and TrepCel4; **c** Enzymatic activity test against CMC

The gene fragments coding for TrepCel3 and TrepCel4 without signal peptide were amplified and ligated into pET-28a (+) vector to construct recombinant plasmids (Fig. 2a). The purification results were checked by SDS-PAGE, and the single TrepCel3 and TrepCel4 bands were observed with a Mw corresponding to the calculated 39.8 kDa and 37.9 kDa, respectively (Fig. 2b). The TrepCel3 and TrepCel4 showed hydrolytic activities against CMC based on the DNS reaction results (Fig. 2c). Therefore, the purified TrepCel3 and TrepCel4 were then subjected to the following biochemical characterization.

### Enzymatic characterization of recombinant enzymes

**Fig. 3:**
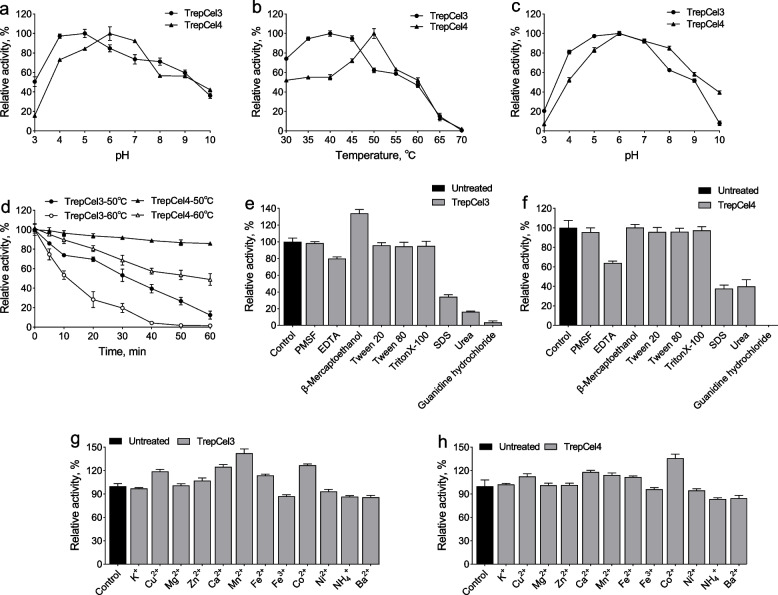
Characterizations of purified recombinant TrepCel3 and TrepCel4. **a** Effect of pH on enzyme activities; **b** Effect of temperature on enzyme activities; **c** pH stabilities of TrepCel3 and TrepCel4; **d** Thermostability assays of TrepCel3 and TrepCel4; **e **and** f** Effect of chemical solutions on the activities of TrepCel3 and TrepCel4; **g **and** h** Effect of metal ions on the activities of TrepCel3 and TrepCel4. All the data values represent the means ± SD

The optimal pH values of TrepaCel3 and TrepCel4 were 5.0 and 6.0, respectively. Two enzymes both exhibited > 50% of their maximal activities at pH 4.0 to 9.0 (Fig. 3a). At the individual optimal pH value, the maximum activity was observed at 40 °C for TrepCel3, and 50 °C for TrepCel4 in the range of temperature between 30 and 70 °C (Fig. 3b). To our best knowledge, only the known acidic endoglucanase Cel-3.1 from buffalo rumen metagenome had low optimum temperature (35 °C) [66]. The optimum pH of TrepCel4 was higher than some reported endoglucanases (mean approximately 5.1, Table 1). Most of endoglucanases hardly had activity over a broad pH range, only the endoglucanase Cel‑5A showed relative high activities from pH 4.0 to 10.0 [67]. Interestingly, the temperature optimum (50 °C) of Cel‑5A was the same as TrepCel4.

**Table 1:** Properties comparison of TrepCel3 and TrepCel4 with microbial endoglucanases of family 5

Protein	Carboxymethyl cellulase (CMCase) activity			References
	Optimum pH/ TM, ℃	*V*_max_, U/mg ^1^	*K*_m_, mg/mL^1^	
AcCel12B	4.5/75	131.8	25.5	[19]
Cel-5M	6/40	27.1 ± 0.08	2.7	[25]
CFA2	4.8/70	556.58	12.0	[68]
GtCel5	6/60	124	9.5 ± 0.9	[69]
ApCel5A	4.5/40	264 ± 40	7.4 ± 1.4	[70]
GtCel12A	4.5/50	101 ± 7	2.6 ± 0.3	[70]
StCel5A	6/70	194 ± 86	11 ± 5	[70]
Cel‑5A	4.5/50	19.19	14.87	[67]
GtCel5	4/70	1475 ± 71	4.5 ± 0.3	[71]
BaGH5‐WT	6/65	6.0 ± 0.6	0.38 ± 0.11	[72]
TrepCel3	5/40	228.3 ± 15.80	3.6 ± 0.43	This study
TrepCel4	6/50	219.7 ± 13.11	2.3 ± 0.38	This study

The TrepCel3 maintained > 20% residual activity after pre-incubating at pH 3.0 to 9.0 for 16 h at 4 °C, but exhibited approximately 7.9% activity at pH 10.0 (Fig. 3c). The TrepCel4 retained > 39% activity at pH 4.0 to 10.0, while had low activity (approximately 7.1%) at pH 3.0 (Fig. 3c). The pH stability of TrepCel4 was similar with the known enzyme counterpart endoglucanase AgCMCase from *Aspergillus glaucus* CCHA that exhibited > 40% residual activity at pH 4.0 to 10.0 [73]. When TrepCel3 was incubated at 50 or 60 °C for 60 min, it lost most of enzymatic activity, suggesting that TrepCel3 was not a thermophilic cellulase (Fig. 3d). In some published studies, endoglucanases like Cel5A-h38 [74], Cel5A-h28 [22], CelEx-BR12 [75] and CMC‑1 [23] were not stable at 50 °C, which was consistent with TrepCel3. The TrepCel4 retained > 85% residual activity at 50 °C for 60 min, and > 50% at 60 °C for 60 min (Fig. 3d), indicating that TrepCel4 may be a thermophilic cellulase. The thermostability of the endoglucases is especially crucial for its putative use in industrial and livestock farming applications. Thermostable endoglucases with higher optimum temperature could improve the economic viability of industrial processes [68].

The effects of a range of metal ions and chemical reagents on enzymatic activities of TrepCel3 and TrepCel4 were measured as shown in Fig. 3e–h. The presence of Cu^2+^, Mg^2+^, Zn^2+^, Ca^2+^, Mn^2+^, Fe^2+^ and Co^2+^ had major influences increasing enzymatic activities of TrepCel3 and TrepCel4, while Fe^3+^, Ni^2+^, NH_4_^+^ and Ba^2+^ decreasing enzymatic activities. For TrepCel3, Mn^2+^ strongly stimulated enzymatic activity (approximately 42%), which was consistent with the endoglucanase Cel-1 from buffalo rumen [24], and the bifunctional enzyme, PersiCelXyn1 from cow rumen [55]. For TrepCel4, in particular, Co^2+^ increased enzymatic activity with approximately 36%, which suggested that Co^2+^ was important for TrepCel3. Similarly, in previous studies, Co^2+^ could also strongly enhance enzymatic activities sun as endoglucanase XacCel8 [56] and EG5C [18]. Different metal ions showed different effects on TrepCel3 and TrepCel4 might due to the differences in their tertiary structures. When PMSF, Tween 20, Tween 80 and TritonX-100 were added, both enzymatic activities of TrepCel3 and TrepCel4 were not affected. The addition of EDTA had inhibitory effects on enzymic activities. The addition of β-mercaptoethanol improved the enzymatic activity of TrepCel3, while did not enhance the activity of TrepCel4. Because, the amino acid sequence of TrepCel3 contained cysteines residues, which was attributed to the reducing effect of β-mercaptoethanol on the disulfide bonds [54], while TrepCel4 did not contain a pair of cysteine residues. Such effects were also observed in some reported studies [38, 76]. The addition of β-mercaptoethanol increased 129% of original activity of the GH5 family cellulase CelDZ1, 29.7% of original activity of CBH6A [38], and 18.4% of original activity of EgGH45 [38]. Furthermore, after the addition of the anionic detergent SDS, TrepCel3 and TrepCel4 still retained approximately 34% and 38% activities, respectively. In textile and paper industries, enzymes applied should be resistant to high temperature and SDS solution [38]. Therefore, TrepCel3 and TrepCel4 might be used in industrial textile processes and products because of their tolerances of surfactants.

### Effects of salt solution on enzymatic activity

**Fig. 4:**
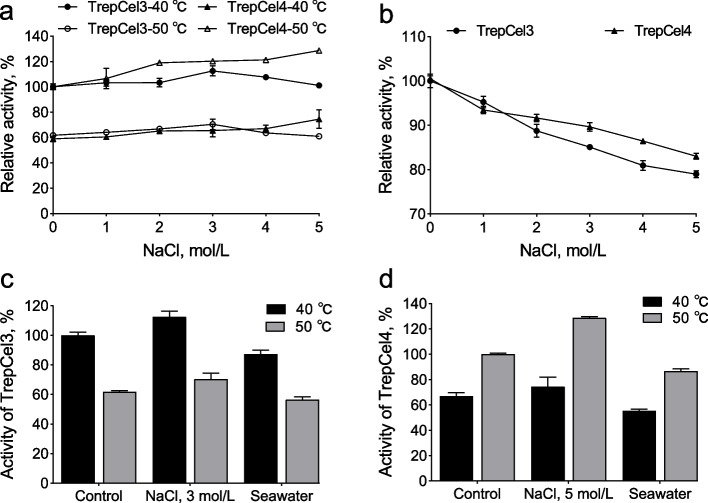
Enzymatic activity of TrepCel3 and TrepCel4 in different salt solutions. **a** Effect of NaCl on the activities of TrepCel3 and TrepCel4; **b** Effect of NaCl on the stabilities of TrepCel3 and TrepCel4; **c** The enzyme activity of TrepCel3 in artificial seawater compared with that in 3 mmol/L NaCl and the salt-free condition. The activity was calculated as relative (%) to the enzyme activity in the salt-free at 40 °C; **d** The enzyme activity of TrepCel4 in artificial seawater compared with that in 5 mmol/L NaCl and the salt-free condition. The activity was calculated as relative (%) to the enzyme activity in the salt-free at 50 °C. All the data values represent the means ± SD

**Fig. 5:**
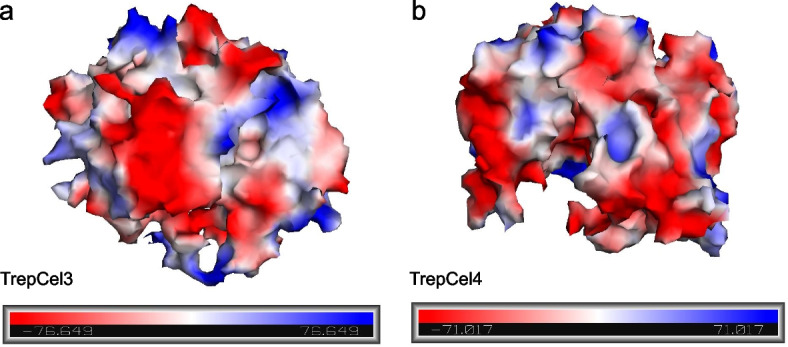
The predicted surface electrostatic potentials of (**a**) TrepCle3 and (**b**) TrepCel4. The negative and positive electrostatic potentials are reflected by red and blue, respectively

Generally, endoglucases with excellent specificities are of worthwhile interest for industrial applications where extremophilic conditions like high temperature and salt concentrations. In addition, for industrial applications, seawater is abundant and amply available in nature, and usually used as an efficient solvent for hydrothermal pretreatment of plant biomass [77]. Enzymatic activities of TrepCel3 and TrepCel4 were both remarkably enhanced by 1 to 5 mol/L NaCl at individual optimum temperature (Fig. 4). With the addition of 3 mol/L NaCl, TrepCel3 had 13% and 9% enhancement as compared with initial activity at 40 and 50 °C, respectively. When TrepCel4 was incubated with 5 mol/L NaCl, it retained 129% of initial activity at 50 ℃, and it had 16% enhancement as compared with control at 40 °C. Hence, the optimal NaCl concentration for maximum stimulation of the TrepCel3 and TrepCel4 activities assayed at 40 and 50 °C were 3 and 5 mol/L, respectively. However, enzymatic activities of TrepCel3 and TrepCel4 were slightly inhibited by 5 mol/L NaCl at 50 and 40 °C, respectively. Notably, the NaCl stimulation at individual optimum temperature could improve the enzymatic activities to higher than the obtainable activity of TrepCel3 at 50 °C, and TrepCel4 at 40 °C. Moreover, TrepCel3 and TrepCel4 obtained more than 83% and 79% enzymatic activities after 60 min at RT respectively. The application potentials of TrepCel3 and TrepCel4 for catalysis in artificial srawater were further evaluated. Enzymatic activities of TrepCel3 and TrepCel4 were both slightly inhibited by artificial seawater compared to controls (Fig. 4c and d). As Fig. 5 shown, TrepCel3 and TrepCel4 had high distributions of acidic amino acids on the surface leading to an overall negative electrostatic potential, which can facilitate the weakening of hydrophobicity or strengthening of hydrophilic forces on enzymatic surface to improve water-binding capacity and stop enzymes aggregation at high salt solution [78, 79].

If possible, cellulolytic enzymes applied in textile industries or biofuel synthesis that should be tolerant to various salts. A novel alkali thermostable endoglucanase PersiCel4 from sheep rumen metagenome exhibited high tolerance to the high concentration of NaCl (5 mmol/L) [80]. However, to our best knowledge, most of published studies just focused on the effect of NaCl solution on xylanase activity. For instance, the activity of xylanase XylCMS from camel rumen was improved with the highest activity enhancement by different NaCl concentrations (1 to 5 mmol/L) especially 3 mmol/L NaCl [57]. Besides, enzymatic activity of XylCMS was significantly stimulated by 77% at 37 °C in artificial seawater as compared with salt free control, while TrepCel3 and TrepCel4 were inhibited in artificial seawater. There are several reasons accounting for this phenomenon: 1) the camel diet mainly consists of low quality and woody lignocellulose, which are not desired by cow, goat, sheep and buffalo; 2) camels are all typically found in desert regions and other extreme harsh environments. Hence, extreme halophilic xylanases from camel rumen are easily found. Additionally, enzymatic activities of some published xylanases were stimulated by low NaCl concentrations (0.5 to 1.5 mmol/L) [81–83], but inhibited in higher NaCl concentrations. Enzymatic activity of alkaline xylanase rXynSL3 from *Alkalibacterium* sp. SL3 was even inhibited by 0.25 mmol/L NaCl [78].

### Substrate specificity and kinetic determinations

**Fig. 6:**
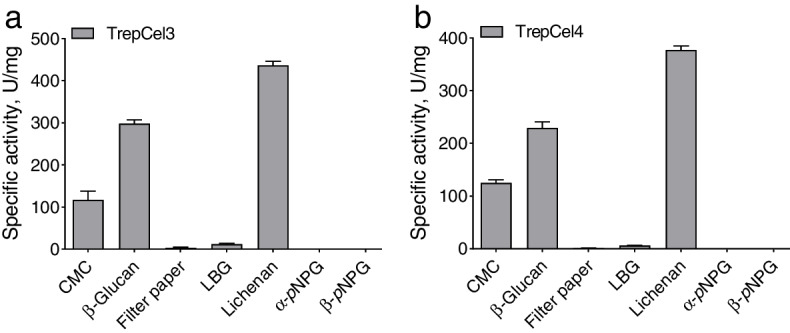
Specific activities of (**a**)TrepCel3 and (**b**) TrepCel4. All the data values represent the means ± SD

The substrate activities were measured in the presence of various substrates. The results of the specific activity assays were shown in Fig. 6a and b, TrepCel3 and TrepCel4 exhibited the highest activity in lichenan 436.9 ± 8.30 (mean ± SD) and 377.6 ± 6.80 U/mg, respectively. Enzymatic activities of TrepCel3 and TrepCel4 against CMC and barely β-glucan were lower than that of lichenan. Two novel thermostable endoglucanases PersiCel1 and PersiCel2 also showed the highest activity in lichenan 773.5 and 636.9 U/mg, respectively, and enzymatic activities of TrepCel3 and TrepCel4 against CMC and barely β-glucan were lower than that of lichenan [31]. But enzymatic activities of TrepCel3 and TrepCel4 against lichenan were lower than that of PersiCel1 and PersiCel2 because of the difference of the third structure between them. The TrepCel3 and TrepCel4 exhibited very little enzymatic activities against filter paper and LBG. In addition, these two cellulases did not show any enzymatic activities against α-*p*NPG and β-*p*NPG. Based on the result assays, TrepCel3 and TrepCel4 had higher ability to hydrolyze CMC, but no ability to hydrolyze *p*NPG, which indicated that TrepCel3 and TrepCel4 were both endoglucanases not β-glucosidases.

The kinetic parameters examined for barely β-glucan, lichenan and CMC were shown in Table 2. The maximal velocity (*V*_max_) values of TrepCel3 for barely β-glucan, lichenan and CMC were 527.3 ± 22.69, 883.8 ± 25.59 and 228.3 ± 15.80 U/mg, respectively. The determined *K*_m_ (Michaelis–Menten constant) values of TrepCel3 for barely β-glucan, lichenan and CMC were 4.9 ± 0.73, 7.1 ± 0.58 and 3.6 ± 0.43 mg/mL. The *V*_max_ values of TrepCel4 for barely β-glucan, lichenan and CMC were 441.4 ± 9.32, 772.3 ± 48.65 and 219.7 ± 13.11 U/mg, respectively. The *K*_m_ values of TrepCel4 for barely β-glucan, lichenan and CMC were 3.9 ± 0.14, 5.0 ± 0.47 and 2.3 ± 0.38 mg/mL, respectively. Compared with some reported studies, TrepCel3 and TrepCel4 had higher *V*_max_ values (CMC as substrate) than enzymes such as AcCel12B [19], GtCel5 [69] and StCel5A [70]. The *K*_m_ values could reflect the affinity of an enzyme towards its various substrate. Based on the *K*_m_ values, TrepCel3 and TrepCel4 both had the highest affinity against CMC instead of β-glucan and lichenan. Besides, *K*_m_ values of TrepCel3 and TrepCel4 (CMC as the substrate) were lower than that of AcCel12B [19], GtCel5 [69] and StCel5A [70], which indicated that TrepCel3 and TrepCel4 had better affinity to CMC than these enzymes that we mentioned above.

**Table 2:** The kinetic parameters of TrepCel3 and TrepCel4

Protein substrate type	Kinetic properties	
	*V*_max_, U/mg	*K*_m_*,* mg/mL
TrepCel3, β-Glucan	527.3 ± 22.69	4.9 ± 0.73
TrepCel3, Lichenan	883.8 ± 25.59	7.1 ± 0.58
TrepCel3, CMC	228.3 ± 15.80	3.6 ± 0.43
TrepCel4, β-Glucan	441.4 ± 9.32	3.9 ± 0.14
TrepCel4, Lichenan	772.3 ± 48.65	5.0 ± 0.47
TrepCel4, CMC	219.7 ± 13.11	2.3 ± 0.38

All the data values represent the means ± SD

### Synergism of TrepCel3 and TrepCel4

**Fig. 7:**
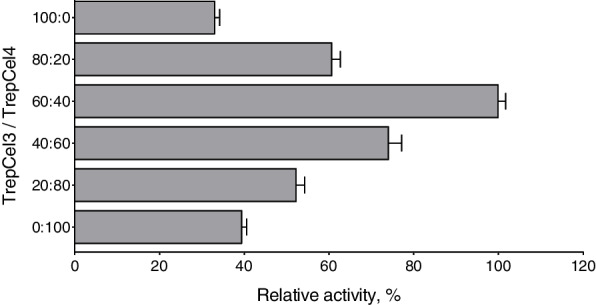
Synergism of TrepCel3 and TrepCel4 in the hydrolysis of CMC substrate

When TrepCel3 and TrepCel4 were incubated together with CMC, a strong synergy effect was observed in line with the production of reducing sugar compared with the released reducing sugar by TrepCel3 and TrepCel4, separately. The combination of TrepCel3 and TrepCel4 at the ratio of 60:40 showed the highest efficiency (Fig. 7). As we mentioned above, enzymatic activity of TrepCel3 against CMC was lower than that of TrepCel4 (Fig. 6), to obtain the best ratio of TrepCel3 and TrepCel4, TrepCel3 may need to account for higher percent. Two thermostable endoglucanases (PersiCel2 and PersiCel1) from camel rumen microbiome samples at the ratio of 60:40 also exhibited the highest efficiency [31].

**Fig. 8:**
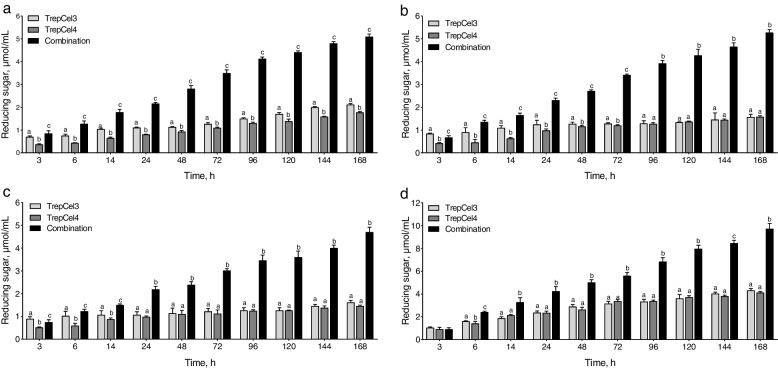
Reducing sugar of hydrolyzed (**a**) rice straw, (**b**) wheat straw, (**c**) leymus chinensis and (**d**) sugar beet pulp by TrepCel3, TrepCel4 and the enzymatic combination (TrepCel 3:TrepCel 4 at 60:40), respectively. All the data values represent the means ± SD

The constituent components in lignocellulosic feedstocks were listed in Additional file 2: Table S1, RS is composed of 42.03% cellulose, while this value is 35.74% for WS, 36.24% for LC, and 22.14% for SBP; RS is composed of 35.31% hemicellulose, while this value is 34.07% for WS, 32.24% for LC, and 15.35% for SBP. As shown in Fig. 8a, for the hydrolysis of RS, in the first 24 h, 1.10, 0.79 and 2.24 μmol/L reducing sugars were generated by TrepCel3, TrepCel4, and the combination of them, respectively, and then reached 2.12, 1.77 and 5.10 μmol/L at 168 h. Total production of reducing sugar of the combination group was significantly higher than that of TrepCel3 and TrepCel4 group at 168 h, separately (*P* < 0.05). Production of reducing sugar of the hydrolysis of WS and LC at 24 and 168 h was similar with RS (Fig. 8b and c). However, for the hydrolysis of SBP, in the first 24 h, 2.36, 2.33 and 4.23 μmol/L reducing sugars were generated by TrepCel3, TrepCel4, and this combination, respectively, and then reached 4.32, 4.12 and 9.72 μmol/L at 168 h. Production of reducing sugar from the hydrolysis of SBP by the combination at 168 h was higher than that of RS, WS and LC (Fig. 8).

As we known that high lignocellulose loadings typically hinder the enzymatic hydrolysis of cellulose. As Additional file 2: Table S1 shown, by comparison, SBP contains relatively lower cellulose, hemicellulose and lignin than RS, WS and LC, this means that SBP is easier to be hydrolyzed by TreCel3, TrepCel4, and the combination of them. We also found that the combination of TrepCel3 and TrepCel4 had higher ability than individual cellulase to hydrolyze SBP. Besides, using WS, RS and LC as the substrates were also observed the similar results. These results suggested the excellent potential of the combination of TrepCel3 and TrepCel4 in converting the lignocellulosic biomass to reducing sugar. Efficient hydrolysis of the natural renewable residues by the combination of different cellulases was also reported in previous studies [31, 38, 84, 85]. The reason is that cellulose is effectively hydrolyzed by the cooperative actions of endoglucanase (EC 3.2.1.4) that randomly cleaves the intramolecular β-1,4glycosidic bonds to generate soluble oligosaccharides, exoglucanase (EC 3.2.1.91) that releases cellobioses from the free ends, and β-glucosidase (EC 3.2.1.21) that hydrolyzes cellobiose to glucose [86]. Different cellulases incubated together in the optimal proportion can overcome individual shortcomings and deficiencies. The combination of TrepCel3 and TrepCel4 could be of interest for industrial and livestock farming applications because of its high hydrolysis efficiency towards agricultural biomass. Therefore, we further subjected RS, WS and LC for enzymatic hydrolysis in in vitro incubation to investigate the effect of this combination on rumen digestion and fermentation parameters.

### In vitro rumen fermentation

As shown in Table 3, after 48 h of incubation, supplementation with the combination of TrepCel3 and TrepCel4 (1.2 mg TrepCel3, 0.8 mg TrepCel4) significantly increased DMD of RS, WS and LC by 4.3%, 4.5%, and 4.8%, respectively, compared with controls (*P* < 0.05). The enzyme treatments showed greater gas production volume of RS, WS and LC by 21.6%, 23.4%, and 15.5%, respectively (*P* < 0.05), while did not affect fermentation fluid pH (*P* > 0.05). The concentrations of TVFAs, acetate, propionate and other VFAs (isobutyric acid, valeric acid, isovaleric acid) were significantly affected by the addition of enzyme (*P* < 0.05), resulting in a significant increase in the acetate:propionate ratio (A:P, *P* < 0.05). When enzyme incubated with RS, butyrate concentration was significantly increased (*P* < 0.05), but when incubated with WS or LC, the butyrate concentration was not significantly affected (*P* > 0.05). Compared to the control, acetate and propionate molar proportions were significantly greater in enzyme-added lignocellulosic residues (*P* < 0.05), while butyrate molar proportion was significantly lower (*P* < 0.05). With RS, the addition of enzymatic combination did not affect the molar proportion of other VFAs (*P* > 0.05), other VFAs were significantly decreased by enzyme in WS and LC (*P* < 0.05). The supplementation of enzyme significantly increased the NH_3_-N concentration of RS and WS group (*P* < 0.05), while did not affect the NH_3_-N concentration of LC group (*P* > 0.05). Application of enzyme significantly increased the MCP concentration of RS, WS and LC group, and the lactate concentration of RS group (*P* < 0.05).

**Table 3:** Effect of enzymatic combination (TrepCel3:TrepCel4 at 60:40) on rumen digestion and fermentation of RS, WS and LC in in vitro microbial fermentation

Item	RS		*P* value	WS		*P* value	LC		*P* value
	Control	Combination		Control	Combination		Control	Combination	
DMD, %	7.2 ± 1.60	11.5 ± 1.25	0.021	7.2 ± 1.61	11.7 ± 1.27	0.019	7.4 ± 1.63	12.2 ± 1.32	0.016
Total gas, mL	83.8 ± 1.61	101.9 ± 0.57	0.004	81.2 ± 4.40	100.2 ± 6.51	0.010	90.7 ± 2.22	104.8 ± 6.69	0.027
pH	7.1 ± 0.07	7.1 ± 0.06	0.219	7.1 ± 0.08	7.2 ± 0.11	0.592	7.1 ± 0.08	7.1 ± 0.07	0.462
NH_3_-N, mmol/L	4.2 ± 0.68	5.8 ± 0.72	< 0.001	3.7 ± 0.64	5.6 ± 1.08	< 0.001	5.4 ± 0.98	5.9 ± 1.25	0.360
MCP, µg/mL	142.0 ± 24.57	170.3 ± 14.70	0.009	141.1 ± 15.10	161.6 ± 8.54	0.003	157.0 ± 18.33	188.5 ± 27.91	0.012
Lactate, mmol/L	2.3 ± 0.15	2.5 ± 0.18	0.020	2.4 ± 0.23	2.5 ± 0.13	0.250	3.3 ± 0.35	3.4 ± 0.28	0.496
TVFA, mmol/L	75.4 ± 2.75	89.1 ± 2.50	0.003	70.9 ± 2.5	84.8 ± 5.23	0.015	76.4 ± 1.27	84.3 ± 0.81	0.001
Acetate	47.8 ± 1.95	57.2 ± 1.80	0.004	44.9 ± 1.49	54.4 ± 3.19	0.010	47.7 ± 0.60	53.5 ± 0.64	< 0.001
Propionate	17.4 ± 0.59	19.5 ± 0.65	0.014	16.3 ± 0.61	18.4 ± 1.09	0.043	17.4 ± 0.44	18.5 ± 0.10	0.014
Butyrate	7.3 ± 0.19	8.2 ± 0.24	0.007	7.0 ± 0.30	8.1 ± 0.62	0.057	7.9 ± 0.28	8.2 ± 0.05	0.262
Other VFAs	2.9 ± 0.07	4.3 ± 0.25	0.001	2.7 ± 0.12	3.9 ± 0.34	0.005	3.4 ± 0.13	4.1 ± 0.04	0.001
Individual, mol/100 mol total VFA									
Acetate	64.2 ± 0.30	63.4 ± 0.44	0.059	64.2 ± 0.23	63.4 ± 0.23	0.01	63.5 ± 0.21	62.4 ± 0.53	0.031
Propionate	21.9 ± 0.21	23.1 ± 0.21	0.002	21.7 ± 0.06	22.9 ± 0.10	< 0.001	22.0 ± 0.10	22.8 ± 0.21	0.003
Butyrate	9.2 ± 0.15	9.6 ± 0.15	0.020	9.5 ± 0.17	9.9 ± 0.10	0.026	9.7 ± 0.06	10.4 ± 0.25	0.009
Other VFAs	4.8 ± 0.36	3.9 ± 0.06	0.011	4.6 ± 0.15	3.8 ± 0.06	0.001	4.9 ± 0.01	4.4 ± 0.15	0.006
A:P ratio	2.8 ± 0.04	2.9 ± 0.02	0.003	2.8 ± 0.02	3.0 ± 0.01	< 0.001	2.7 ± 0.05	2.9 ± 0.02	0.007

Rice straw, RS; wheat straw, WS; leymus chinensis, LC; dry matter digestibility, DMD; total volatile fatty acid, TVFA; acetate: propionate ratio, A:P ratio;

Other VFAs, isobutyric acid, valeric acid, and isovaleric acid; microbial protein, MCP. *P*-values were calculated using *t*-test. All the data values represent the means ± SD

The EFEs can increase the nutrient digestibility of agricultural residues because it can disrupt the basic structure of straw fiber [61], improve the growth of rumen bacteria [87], and stimulate bacterial attachment [88]. Enzymatic combination of TrepCel3 and TrepCel4 significantly increased the DMD, which was consistent with the results reported by Giraldo et al. [89]. Briefly, the cellulase combination (a cellulase from *Trichoderma longibrachiatum* and a cellulase from *Aspergillus niger*) could significantly improve the in vitro fermentation parameters of diet (70% grass hay and 30% concentrate, DM basis) including NDF and ADF apparent digestibility [89]. Gas production in vitro is an indirect measurement of lignocellulosic substrate degradation, and Wallace et al. [90] presented that there was a significant positive correlation between enzymatic activity and gas production in vitro from grass silage. The relatively higher enzymatic activity can increase the rate of in vitro gas production from corn silage [90]. In this study, the addition of enzymatic combination significantly increased the in vitro gas production, but the relationship between gradient dose of enzyme and gas production was not investigated. It is not ignored that the relatively higher dose of EFEs may prevent the binding of enzymes to lignocellulosic substrate receptors, which hinder the proportional attachment by bacteria to straw fiber [91]. Current results showed that enzymatic combination supplementation had no effect on fermentation fluid pH value. The rumen fluid pH is one of the important factors affecting the straw fiber decomposition because rumen bacteria is sensitive to low pH value [92]. The neutral pH of this study, indicating that these two endoglucanases did not have negative effects on fibrolytic bacteria. Normally, the rumen NH_3_-N concentration is range from 7.7 to 17.3 mmol/L, which is regarded as N source for MCP synthesis [93]. However, current results showed that the NH_3_-N is relatively low because of the low crude protein content of straw fiber. Supplemental the enzyme also increased the production of NH_3_-N for RS and WS. Vallejo et al. [7] presented that feeding sheep on basic diet treated with the enzyme (XY6 and XY3) improved the NH_3_-N concentration compared with the control group. The quantity of dietary nitrogen breakdown and nitrogen absorption by ruminal bacteria may be responsible for the increased generation of NH_3_-N [94]. The carbohydrates that are readily accessible and the nitrogen that is digested in the rumen are both necessary for MCP production. Higher MCP concentration because of increasing in the fermentable DM were also found in previous studies [10, 61], which was also similar with our results. The acetate, propionate and TVFA production were significantly increased by the enzyme regardless of straws, which was consistent with the results reported by Giraldo et al. [89]. Briefly, a cellulase combination (a cellulase from *Trichoderma longibrachiatum* and a cellulase from *Aspergillus niger*) could significantly improve VFA production [89]. Supplemental TrepCel3 and TrepCel4 not only increased acetate, propionate and butyrate concentrations, but also increased their molar proportions. Interestingly, although the addition of enzymatic combination increased other VFAs concentration, the molar proportion of Other VFAs was decreased. The increased acetate and butyrate concentrations with EFEs addition can be enhanced decomposition of structural carbohydrates [95]. In the rumen, structural carbohydrates fermentation rather than starch, could stimulate the growth of fibrolytic rumen bacteria, and yielded more acetate relative to starch [96]. Li et al. [10] found that adding EFEs significantly increased the relative abundances of acetate producing bacteria such as the phylum of Firmicutes and genus of *Desulfovibrio*. However, in this study, we did not investigate the change of fibrolytic bacterial community. Rumen butyrate is an energy source or metabolized to β-hydroxybutyrate by rumen epithelial cells, and it can also be regarded as a primer for short fatty acid synthesis [97]. Butyrate is absorbed through the rumen wall and can be used as a primer for short- and even-chain milk fatty acid synthesis [97]. As we all know that, propionate is the only major VFA that contributes to glucose synthesis, while the A:P ratio is significantly higher of enzyme-added group of this study, indicating that the addition of TrepCel3 and TrepCel4 lead to a shift in the metabolic pathways. Eun et al. [98] also found that adding EFEs reduced A:P compared with untreated group. We guess that the differences in EFEs and the types or length of lignocellulosic substrates may result in these differences.

## Conclusions

In this study, two novel endoglucanases from buffalo rumen metagenome were successfully cloned, expressed, and characterized. When incubated with Mn^2+^ and Co^2+^ ions (10 mmol/L), degradation efficiencies of TrepCel3 and TrepCel4 increased, respectively. TrepCel3 and TrepCel4 were active at pH 5.0 and 6.0, respectively. Enzymatic activities of TrepCel3 and TrepCle4 could be improved by 3 and 5 mol/L NaCl, respectively. Compared to TrepCel3 and TrepCel4 separately, the combination of them was capable of producing high concentrations of reducing sugar from agricultural feedstocks. The enzymatic combination of TrepCel3 and TrepCel4 gave the highest efficiency at the ratio of 60:40. This enzymatic combination improved in vitro rumen fermentation of natural lignocellulosic feedstocks by increasing the DMD, VFA, NH_3_-N and lactate production, and microbial protein synthesis. This enzymatic combination has the potential to be a feed additive to improve agricultural straw utilization in biotechnological and livestock farming applications.

## Supplementary Information

**Additional file 1: Fig. S1a.** The predicted secondary structure of TrepCel3. **Fig. S1b.** The predicted secondary structure of TrepCel4.

**Additional file 2: Table S1.** The constituent components in rice straw, wheat straw, leymus chinensis and sugar beet pulp.

## Data Availability

The metagenomic data was submitted to the NCBI database as the BioProject: PRJNA815894. The sequences of TrepCel3 and TrepCel4 can be obtained from the NCBI under accession numbers OM986951 and OM986952, respectively.

## Abbreviations

ADF: Acid detergent fiber
A:P: Acetate:propionate ratio
CS: Corn straw
CMC: Carboxymethylcellulose
DNS: 3,5-Dinitrosalicylic acid
DM: Dry matter
DMI: Dry matter intake
DMD: Dry matter digestibility
EFEs: Fibrolytic enzymes
*E.** coli*: *Escherichia coli*
EDTA: Ethylenediaminetetraacetic acid
LBG: Locust bean gum
LC: Leymus chinensis
MCP: Microbial protein
Mw: Molecular weight
NDF: Neutral detergent fiber
PMSF: Phenylmethanesulfonyl fluoride
pI: Isoelectric point
*p*NPG: *p*-Nitrophenyl-β-*D*-glucopyranoside
RT: Room temperature
RS: Rice straw
SBP: Sugar beet pulp
SDS: Sodium dodecyl sulfate
TVFA: Total volatile fatty acids
VFA: Volatile fatty acid
WS: Wheat straw
