# Supplementary material for: Production, purification, characterization and application of two novel endoglucanases from buffalo rumen metagenome
Source: J Anim Sci Biotechnol. 2023 Feb 6;14:16. doi: 10.1186/s40104-022-00814-z (PMC9900955; doi:10.1186/s40104-022-00814-z)
Supplement: Supplementary file 1 — Additional file 1: Fig. S1a. The predicted secondary structure of TrepCel3. Fig. S1b. The predicted secondary structure of TrepCel4. [file 40104_2022_814_MOESM1_ESM.docx]

**Additional file 1**


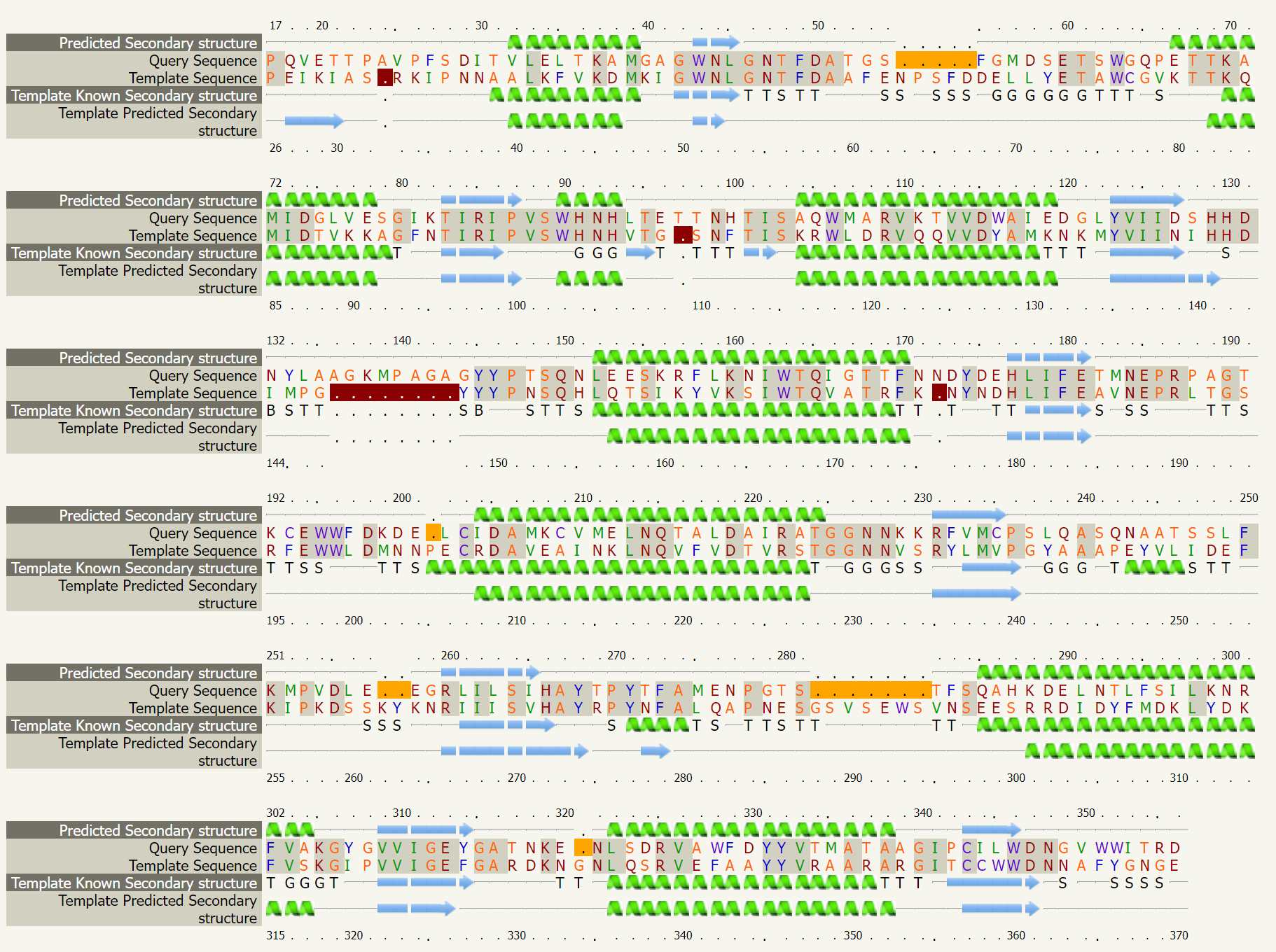


**Fig. S1a** The predicted secondary structure of TrepCel3


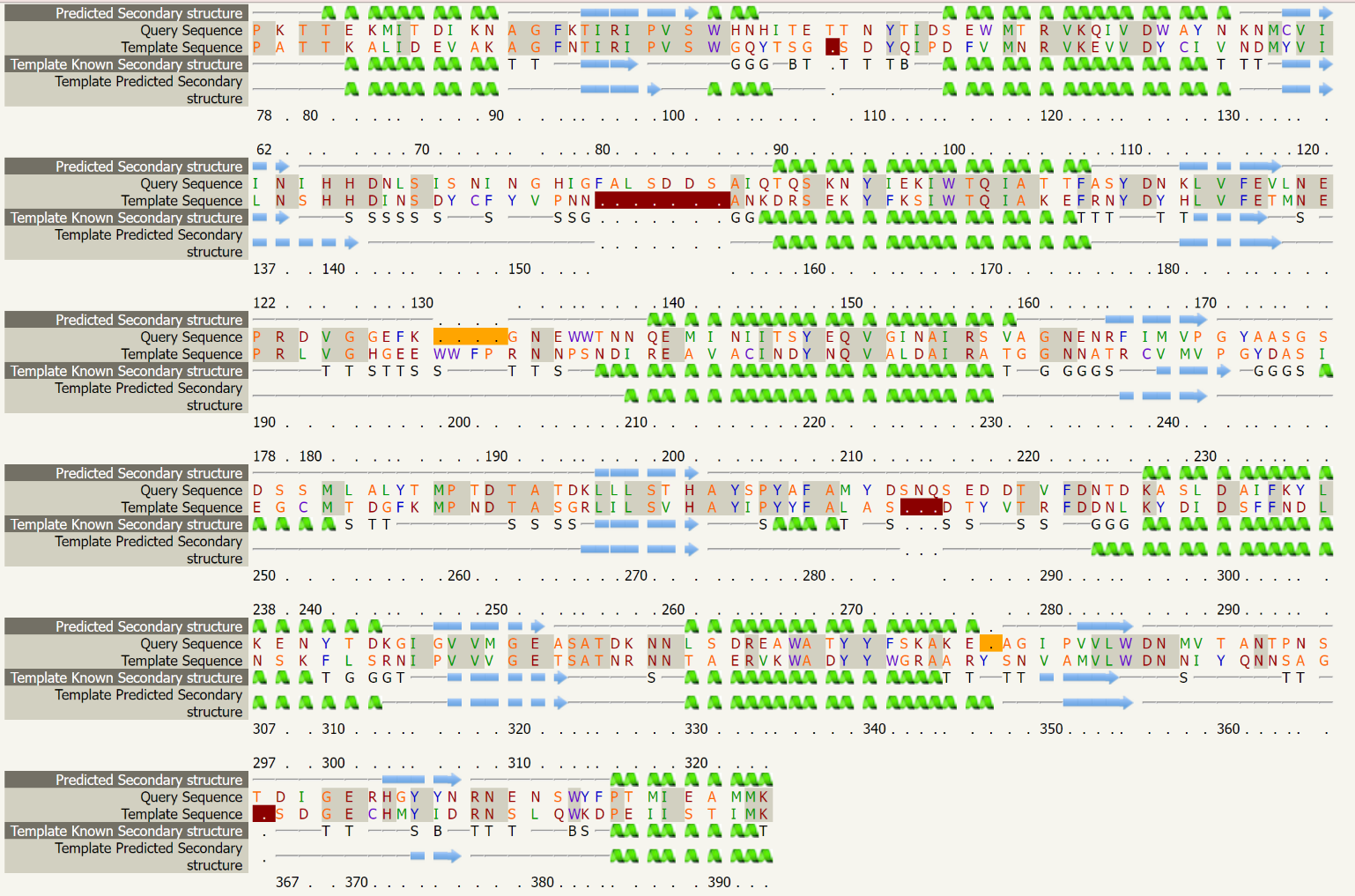


**Fig. S1b** The predicted secondary structure of TrepCel4
