# Supplementary material for: Production, purification, characterization and application of two novel endoglucanases from buffalo rumen metagenome
Source: J Anim Sci Biotechnol. 2023 Feb 6;14:16. doi: 10.1186/s40104-022-00814-z (PMC9900955; doi:10.1186/s40104-022-00814-z)
Supplement: Supplementary file 2 — Additional file 2: Table S1. The constituent components in rice straw, wheat straw, leymus chinensis and sugar beet pulp. [file 40104_2022_814_MOESM2_ESM.docx]

**Additional file 2: Table S1** The constituent components in rice straw, wheat straw, leymus chinensis and sugar beet pulp

| **Biomass type**  **(%w/w, DM)** | **Wheat straw** | **Rice straw** | **Leymus chinensis** | **Sugar beet pulp** |
| --- | --- | --- | --- | --- |
| Cellulose | 35.74±0.95 | 42.03±0.82 | 36.24±0.75 | 22.14±0.18 |
| Hemicellulose | 34.07±0.40 | 35.31±0.79 | 32.24±0.71 | 15.35±0.09 |
| Lignin | 5.77±0.93 | 2.61±0.61 | 6.25±0.77 | 2.33±0.51 |
| NDF | 76.65±0.48 | 81.24±1.06 | 74.86±0.71 | 40.84±0.17 |
| ADF | 42.58±0.19 | 45.93±0.36 | 42.62±0.00 | 25.49±0.11 |
| NDS | 23.35±0.48 | 18.76±1.06 | 25.14±0.71 | 59.16±0.17 |

All the data values represent the means ± SD
